# High-Throughput Omics Technologies: Potential Tools for the Investigation of Influences of EMF on Biological Systems

**DOI:** 10.2174/138920209787847050

**Published:** 2009-04

**Authors:** M Blankenburg, L Haberland, H.-D Elvers, C Tannert, B Jandrig

**Affiliations:** 1 Department of Bioethics & Science Communication, Max-Delbrück-Center for Molecular Medicine Berlin-Buch, 13092 Berlin, Germany;; 2 Present address: Center for Prenatal Diagnostics, 10719 Berlin, Germany;; 3 Present address: FGF, The Research Association for Radio Applications, 53111 Bonn, Germany; 4 Present address: Institute of Sociology, Technical University Dresden, 01062 Dresden, Germany

**Keywords:** RF-EMF, omics, transcriptomics, ionising radiation.

## Abstract

The mode of action of a huge amount of agents on biological systems is still unknown. One example where more questions than answers exist is covered by the term electromagnetic fields (EMF). Use of wireless communication, e.g. mobile phones, has been escalated in the last few years. Due to this fact, a lot of discussions dealt with health consequences of EMF emitted by these devices and led to an increased investigation of their effects to biological systems, mainly by using traditional methods. Omics technologies have the advantage to contain methods for investigations on DNA-, RNA- and protein level as well as changes in the metabolism.

This literature survey is an overview of the available scientific publications regarding biological and health effects of EMF and the application of new high-throughput technologies. The aim of the study was to analyse the amount and the distribution of these technologies and to evaluate their relevance to the risk analysis of EMF. At present, only transcriptomics is able to analyse almost all of the specific molecules. In comparison to ionising radiation, fewer articles dealt with health effects of EMF. Interestingly, most of the EMF articles came from European institutions.

Although omics techniques allow exact and simultaneous examinations of thousands of genes, proteins and metabolites in high-throughput technologies, it will be an absolute prerequisite to use standardised protocols and to independently validate the results for comparability and eventually for sound standing statements concerning possible effects of agents like EMF on biological systems.

## INTRODUCTION

The current nomenclature of omics sciences includes genomics for DNA variations, transcriptomics for messenger RNA, proteomics for peptides and proteins, and metabolomics for intermediate products of metabolism [1, 2]. Technological breakthroughs allow simultaneous examination of thousands of genes, transcripts, proteins, and metabolites with high-throughput techniques and analytical tools to extract information. Hypothesis-driven research and discovery-driven research (through omic methodologies) are complementary and synergistic. Modern screening technologies speed up the discovery process and give broader insight into biochemical events that follow the exposure to harmful agents e.g. chemical substances, ionising radiation or electromagnetic fields.

High-throughput transcriptomic and proteomic technologies have been already adopted to study the expression of certain genes and proteins in response to ionising radiation or electromagnetic fields.

The aim of the work is to critically compare the amount of distribution of these omics technologies in the investigations of biological influences of ionising radiation and non-ionising electromagnetic fields (EMF). Therefore, an overview was created containing the used omics methods, their geographical and chronological distribution and the investigated biological endpoints.

For several decades the effects of EMF on the health of humans have been investigated by using traditional methods, and despite intensive research there is still a controversial scientific discussion regarding potential effects in the range below and near existing safety limits. Therefore, electromagnetic fields have been given an increased attention in this study.

To simplify matters, EMF are divided in low frequency (ELF - extremely low frequency; up to 100 kHz) and high frequency (RF - radiofrequency; 100 kHz - 300 GHz) fields in this survey. The latter contain fields of 300 MHz - 300 GHz often defined as microwaves. In technical ways, they are used e.g. by TV transmitters, mobile phones, microwave ovens and radar systems.

The mode of action of low and high frequency electromagnetic fields on biological systems are different: whereas ELF-EMF mainly exert influences on the membrane and thus are capable to stimulate nerves and muscles [3, 4], RF-EMF cause primarily tissue heating by absorption of electromagnetic waves in polar molecules - above all - water [5, 6].

Often ionising radiation (mainly γ-, X-, or UV-radiation) is used as positive control or as co-exposure to look for additive/synergistic effects to non-ionising EMF. Biological effects of ionising radiation are rather well-known, at least in comparison to low-level non-ionising radiation. Additionally, a dose-response relationship is established for the acute effects of ionising radiation.

Omics technologies have already been used in the past few years to clarify the question whether EMF have biological and health consequences. Diverse experimental approaches were utilised to analyse gene and protein expressions in different pathways. In this respect it is important to prove how far the implemented experimental methods are comparable and to what extent they are validated by alternative methods.

The outcome is a recommendation in which direction a possible standardisation in terms of methods and analysis may be possible in order to ensure the comparability of the results and a high quality of omics studies.

## METHODS AND RESULTS

### Selection of Relevant Publications

To collect all relevant publications the databases PubMed-Medline [7], and the EMF-Portal of the Research Centre for Bioelectromagnetic Interaction of the University Hospital of the RWTH Aachen [8] were analysed. PubMed-Medline is a service of the U.S. National Library of Medicine and the National Institutes of Health. The EMF-Portal is a literature database providing summaries of scientific studies, and additional comprehensive information material on the topic “electromagnetic fields and health”.

First scientific reflections relating biological effects of EMF using omics technologies were published in 1998. On this account the observed period of the literature survey comprises the years 1998 to June 2008.

On the basis of specific keywords (genomics/methylation/mutation/array, transcriptomics/expression/mRNA/array, proteomics/expression/protein/array, metabolomics/metabolism/array) scientific articles were selected inside the mentioned databases. In parallel, publications were captured investigating biological effects not only from EMF (ELF-EMF/RF-EMF/electromagnetic field), but also from chemicals and pharmaceuticals, carcinogens and ionising radiation (γ-, X-, or UV), respectively.

In addition to the name of the journal, date of publication, title of the scientific article, and the institute all data dealing with the experimental work were captured (investigated biological material, kind and duration of radiation, used omics technologies, arrays and chips and their manufacturers, validation of the results *via *other methods as well as information concerning the biological effects).

### Much Less Articles Concerning RF-EMF and Omics Technologies Compared to other Areas

When using omics technologies as selection Fig. (**1**) clearly shows more activities in the areas dealing with chemicals and pharmaceuticals than in EMF research.

This is mainly due to the different number of investigated agents. Whereas in the EMF field only a limited number of frequencies were screened there are considerably more chemical substances under testing. In addition, activities like the Toxic Substances Control Act (TSCA) in the US [9] or Registration, Evaluation and Authorization of Chemicals (REACH) [10] of the EU put more attention on the effects of chemicals than of physical items. Not least, the much higher amount of money is to mention that chemical and pharmaceutical companies are spending for testing their products.

### Relatively Few Articles Meet the Prerequisite to Deal with Omics Technologies

**Fig. (2) Revised number of publications.**
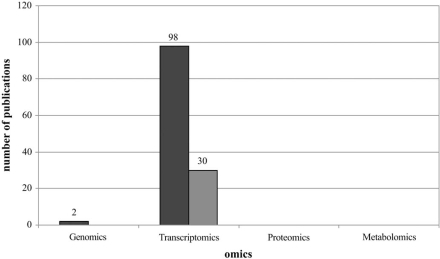
Different shades denote investigated radiation and EMF, respectively (dark grey – ionising radiation, middle grey – EMF).

For the final evaluation, scientific publications concerning EMF and ionising radiation were analysed only if they were written in English, if they were peer reviewed and if the used methods belong to omics technologies. Articles were excluded in the case when only few genes were tested (genomics), few expression data were collected (transcriptomics, proteomics) or few metabolites were analysed (metabolomics). This led to a sharply reduced and changed number of scientific articles (Fig. **2**).

It was obvious that at the present state the promise to analyse almost all of the molecule species can only be met in the transcriptomics field. Only articles with expression analyses at the mRNA level by microarrays were covering a sufficient number of molecules and can be called to use high-throughput omics methods.

### Preferences for Special Manufacturers

**Fig. (3) Most used producers of chips and arrays.**
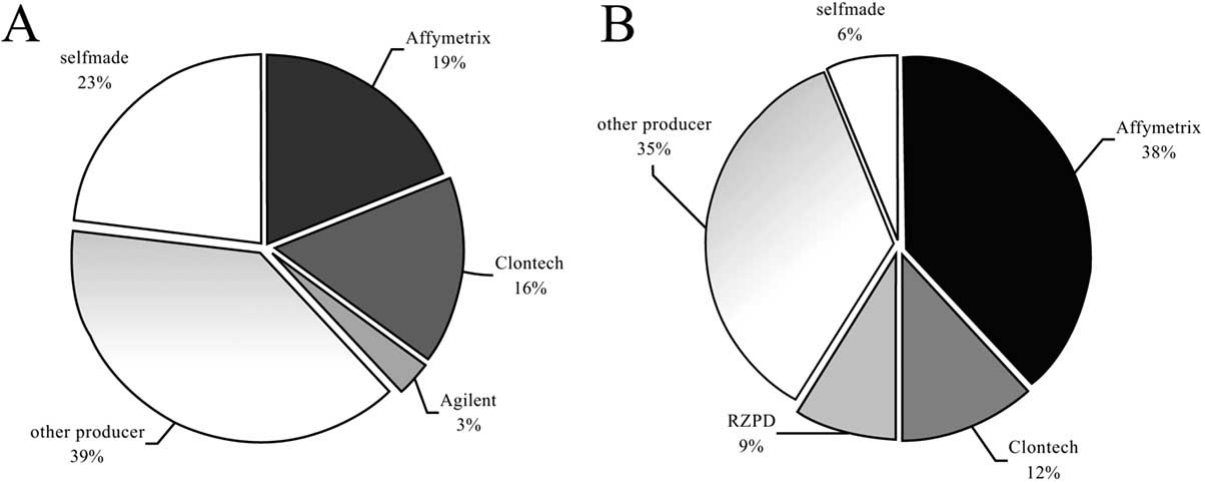
Different colors mark the producer of the most frequently used arrays and chips: **3A** investigation of the effects of ionising radiation and **3B** research on influences of EMF. Producers with lower participation are summarized in term “other”.

Many producers offer a variety of arrays and chips that can be used for omics investigations. In the beginning of the studies often self-made arrays were used. In the experimental work relating to effects caused by ionising radiation (Fig. **3A**), a great diversity is seen.

Products of the companies Affymetrix (19%) and Clontech (16%) were used in about a third of the articles. However, self-made arrays or chips (23%) or products from other producers (39%) were used to a greater extent. This is one reason why it is so difficult to compare and valuate the results from different laboratories.

Interestingly, differences were detected in publications dealing with EMF in comparison to the situation concerning ionising radiation. As seen in Fig. (**3B**) Affymetrix chips were used most (38%). In addition, use of Clontech products (12%) and arrays of the German Resource Centre for Genome Research – RZPD (9%) were described. Nevertheless, self-made arrays of separate labs and diverse arrays of other producers were used in about half of the articles.

However, in the course of time it seems that there exists a clear preference for a limited number of products from few manufacturers.

### Much More Articles Concerning Ionising Radiation and Omics Technologies Compared to EMF

**Fig. (4) Number of publications and the investigated radiations.**
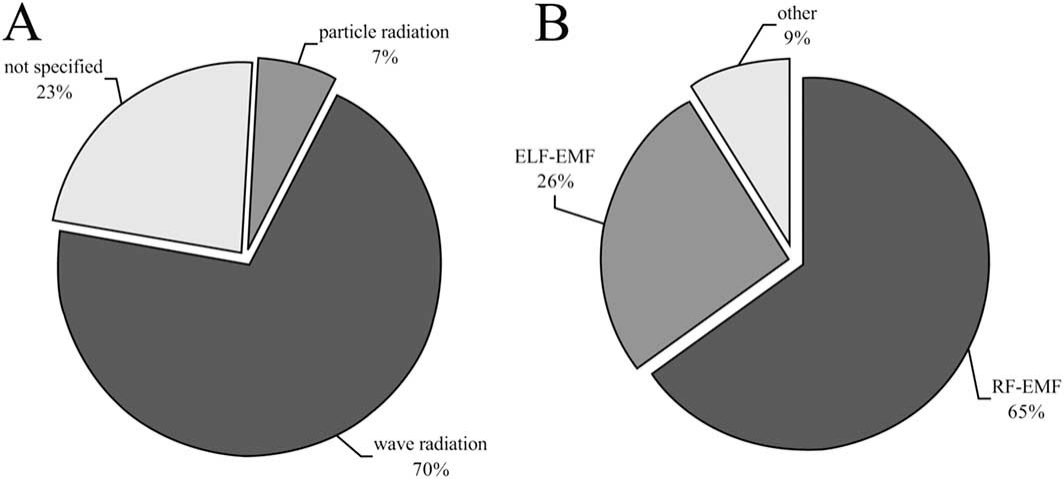
**4A** contains the distribution of the publications belonging to one kind of ionising radiation (dark grey – wave radiation (γ, X and UV), middle grey – particle radiation (α, β, neutrons, HZE), light grey – type of radiation not specified **4B** show the distribution to the different kinds of EMF: dark grey – RF-EMF, middle grey – ELF-EMF, light grey – other).

Articles dealt with ionising radiation mainly investigated particle- and wave radiation (Fig. **4A**).

Effects of wave radiation (containing γ-, X- und UV-radiation) were investigated in 70% of the selected publications. Particle radiation (containing α, β, and neutron radiation as well as HZE - high Z and high energy particle of the galactic cosmic radiation) was studied in 7% of all cases. In 23% of the scientific articles the kind of radiation was not specified.

In the EMF area the most frequently published articles dealt with RF-EMF (65%) (Fig. **4B**). In contrast only 26% of the articles investigated effects of ELF-EMF and 9% of other kind of fields (direct current, 0 Hz), respectively.

**Fig. (5) Number of publications per year.**
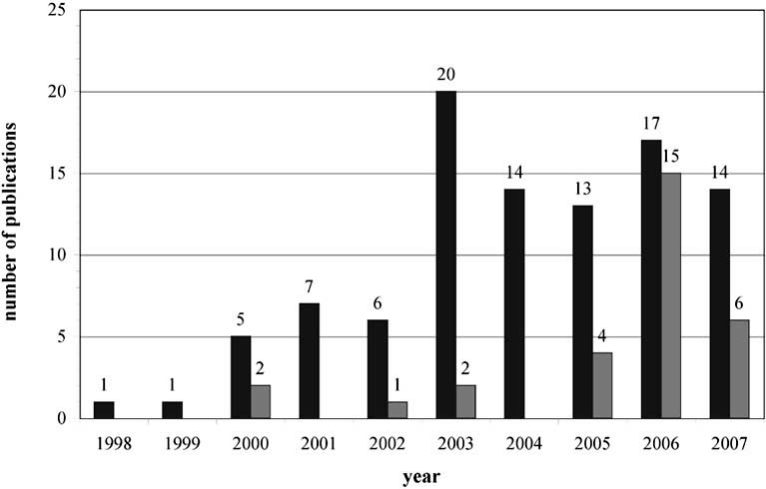
Graded grey shades mark the number of publications containing ionising radiation and EMF in the respective years. (dark grey – ionising radiation, light grey – EMF).

The use of omics technologies in the research on effects from both ionising radiation and EMF occurred in a rather moderate extent (Fig. **5**).

Interestingly, in the first seven years the research on effects of EMF remained on a constant and very low level. After this period of time a short-term but significant increase was detected in 2006. In comparison, the research on ionising radiation showed a constant increase until 2003. A second small maximum can be found in 2006. In 2007, the number of investigations from both, ionising radiation and EMF, fell down remarkably. Articles published in 2008 could not be included in this diagram because data for the year 2008 are fragmentary and could lead to false impressions.

### EMF Research with Omics Methods is Mainly Done in Europe

Fig. (**6**) shows the distribution of the publications per continent.

Laboratories in America, and especially in the US, were very active in investigating health effects of ionising radiation by omics methods. More than half of the articles were published by American authors. In contrast, we observed a nearly equal intensity of research on EMF with omics methods in America, Asia and Europe. It is remarkable that there is such a relatively low number of articles published by American authors. In addition, it is surprising that in comparison to other continents most of the EMF articles came from European institutions.

### High Diversity of Used Biological Materials

An enormous heterogeneity was detected with respect to the investigated biological materials in EMF research as well as in the research of ionising radiation with omics methods (Table **1**).

This diversity makes it even more difficult to compare data from different articles and to appraise whether the detected effects have health consequences to biological systems or not.

## DISCUSSION

This literature survey focused its investigation on volume and distribution of omics technologies and their relevance for risk assessment of EMF in comparison to results in other areas. Although there is an accentuated role of EMF in the media [11] this is not reflected by the number of investigations using modern technologies. A prominent reason could be the lack of knowledge of a clear mode of action of low-intensity EMF and especially of RF-EMF.

Omics technologies allow a fast and - with rising use – cost-effective screening of gene- and protein expression, characterization of new gene- and protein functions, the classification of genes, proteins and metabolites in pathways as well as the identification of therapeutic targets in the organism [12-14]. However, on closer inspection the majority of the investigated articles only used the omics vocabulary but could not fulfil their promises to use high-throughput omics technologies. Published so far only expression analyses at the RNA level using chip arrays came into consideration.

Currently numerous manufacturers of genechips, protein- and antibody arrays exist. Due to different hybridisation technologies of cDNA or oligonucleotides and proteins or antibodies fixed on carrier material [15-18] a direct comparison of results from applications of different manufacturers is very difficult. Nevertheless, cross-platform utilization of gene expression data could reduce the need for duplicate experiments and facilitate a more extensive exchange of data within the research community. Some measures for the correspondence of the results from different gene expression platforms are already developed [19]. Meanwhile, platforms based on different technology principles reveal similar aberration patterns, although unique amplification or deletion peaks at various locations could be detected by one of the platforms only [20].

In our literature survey, self-made arrays and arrays from small manufacturers were used in more than half of all articles. A comparison or validation of the data would be very difficult because in most of the articles modern demands of state of the art experiments are not fulfilled. For instance, to ensure getting reliable data it is essential to perform replicates and control experiments with alternative methods. Both prerequisites were practiced only in about half of the scientific articles.

In comparison to research on ionising radiation it can be seen that in EMF research the usage of Affymetrix arrays has been nearly duplicated. This may be based on the fact that EMF research applying omics technologies started later than the research on ionising radiation. Most probably EMF research benefited from the experience in the research on ionising radiation. In addition, the different numbers of hybridized fragments on arrays of the different manufacturers and/or costs of the gene chips may have been accounted for a preference for one of the companies.

The effects of EMF have been examined since the 1970^th^ years [21, 22]. The increasing use of mobile phones and other wireless telecommunication devices in the last years required a worldwide network of fixed antenna or base stations. Hence, the result was an increased public interest to the induced health effects. This fact is supported by the appearance of the RF-EMF investigations from 1999 to 2007, using omics methods detected in our literature survey.

Approximately in the same time as the chip technology for omics investigations was developed in the 1990^th^ years [23] the World Health Organisation (WHO) created a project (the International Electromagnetic Fields Project) to investigate health risks associated with technologies emitting EMF [24, 25]. In 2003 the WHO classified RF-EMF as an area of research with “high priority” and the European Commission launched the Coordination Action EMF-NET [26]. The research was also financially supported by European projects such as “Risk evaluation of potential environmental hazards from low-energy electromagnetic field exposure using sensitive *in vitro* methods (REFLEX)” which included target-oriented omics methods [27]. This was accompanied by a number of other international, European and national research projects [28]. The intensive financial funding of this research in Europe is reflected in the increased number of scientific articles in EMF research using omics methods in comparison to publications of this research from Asia or America [29]. In 2006 a clear rise of publications in EMF research and investigations of ionising radiation with omics technologies is detected. Most of the projects run in the period from 2000 to 2004 [30]. Therefore, the increase of the references in 2006 seems to be based on the finalization of these research activities. The following drop of articles in 2007 might be due to less funding and that no clear effects of RF-EMF could be found.

## CONCLUDING REMARKS

With the help of omics technologies it is possible to investigate cellular states as well as biological correlations in cell lines, primary tissues and the whole organism. Omics technologies allow economical material usage, the simultaneous investigation of thousand of genes, proteins or metabolites and can be a capable tool to investigate effects of EMF. The data of our literature survey show a very heterogeneous distribution. Almost no material (cells or cell lines) was analysed twice by different groups. In addition, the validation of the omics experiments by other methods is absolute necessary. A sufficient number of replications in the same and in other labs are essential. Up to now in most of the investigations in EMF research using omics technologies this has not been done. Therefore, the data volume of the separate experiments with diverse biological material is not sufficient to give clear scientific statements.

## Figures and Tables

**Fig. (1) Different investigation areas and number of publications.**
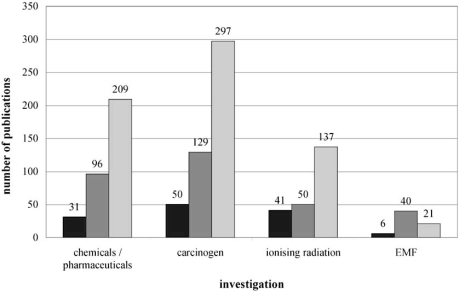
The bars show the number of published scientific articles. The different colors mark applied omics technologies (dark grey – genomics, middle grey – transcriptomics, light grey – proteomics).

**Fig. (6) Number of publications per continent.**
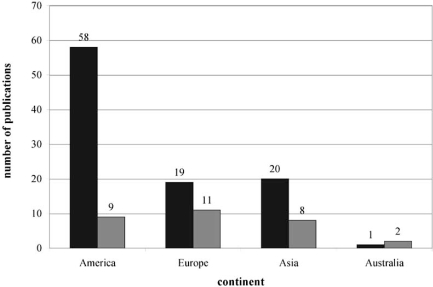
The graphical representation shows the number of publications per continent. To relate the numbers of scientific articles respective to the countries the attribution of the articles to the countries took place by the first author of the publication. The two grey shades mark the considered ionising radiation and EMF, respectively (dark grey – ionising radiation, light grey – EMF).

**Table 1:** Investigated Biological Material: Cell Lines, Tissue of an Organ or Cell Structure are Summarized to One Notation

Biological Material	Number of Publications	
	Ionising Radiation	EMF
connective tissue	12	4
blood cells / ~vessels	24	16
liver	2	1
breast	5	6
lymph	1	0
nerves	3	7
spleen	2	0
cervix / ovar	6	0
lung	2	0
thyroid	4	0
esophagus	3	0
prostate	1	0
skin	4	0
brain	1	0
eye	1	0
tissues / cells of animals*	25	6
plants	2	0
bacteria	3	1
yeast	6	1
hybrid cells	2	0
not specified	2	0
